# Supplementary material for: End Sequence Analysis Toolkit (ESAT) expands the extractable information from single-cell RNA-seq data
Source: Genome Res. 2016 Oct;26(10):1397–410. doi: 10.1101/gr.207902.116 (PMC5052061; doi:10.1101/gr.207902.116)
Supplement: Supplemental Material [file supp_gr.207902.116_Supp_Materials.tar.gz › ESAT-esat_v0.1/doc/html/interfacebroad_1_1pda_1_1seq_1_1segmentation_1_1_read_count_normalizer.html]

ESAT: broad.pda.seq.segmentation.ReadCountNormalizer Interface Reference


ESAT


- Main Page
- Related Pages
- Packages
- Classes
- Files

- Class List
- Class Index
- Class Hierarchy
- Class Members

All Classes Namespaces Files Functions Variables Enumerator Pages

- broad
- pda
- seq
- segmentation
- ReadCountNormalizer

Public Member Functions |
List of all members

broad.pda.seq.segmentation.ReadCountNormalizer Interface Reference

Inheritance diagram for broad.pda.seq.segmentation.ReadCountNormalizer:

[legend]

|  |  |
| --- | --- |
| Public Member Functions | |
| double | normalize (double count) |
|  | |

## Detailed Description

Implements a strategy for read count normalization, so that counts estimated from a given alignment can be compared to counts estimated in a different alignment.

Author
:   mgarber

## Member Function Documentation

|  |  |  |  |  |  |
| --- | --- | --- | --- | --- | --- |
| double broad.pda.seq.segmentation.ReadCountNormalizer.normalize | ( | double | *count* | ) |  |

Normalizes a given read count

Parameters
:   |  |  |
    | --- | --- |
    | count | – raw count estimated from this data model or alignment |

Returns
:   normalized count

---

The documentation for this interface was generated from the following file:

- java/broad/pda/seq/segmentation/ReadCountNormalizer.java


---

Generated on Fri Aug 15 2014 15:11:05 for ESAT by  

 1.8.7
